# Gene–Environment Interactions at Nucleotide Resolution

**DOI:** 10.1371/journal.pgen.1001144

**Published:** 2010-09-30

**Authors:** Justin Gerke, Kim Lorenz, Shelina Ramnarine, Barak Cohen

**Affiliations:** Department of Genetics, Washington University School of Medicine, St. Louis, Missouri, United States of America; North Carolina State University, United States of America

## Abstract

Interactions among genes and the environment are a common source of phenotypic variation. To characterize the interplay between genetics and the environment at single nucleotide resolution, we quantified the genetic and environmental interactions of four quantitative trait nucleotides (QTN) that govern yeast sporulation efficiency. We first constructed a panel of strains that together carry all 32 possible combinations of the 4 QTN genotypes in 2 distinct genetic backgrounds. We then measured the sporulation efficiencies of these 32 strains across 8 controlled environments. This dataset shows that variation in sporulation efficiency is shaped largely by genetic and environmental interactions. We find clear examples of QTN:environment, QTN: background, and environment:background interactions. However, we find no QTN:QTN interactions that occur consistently across the entire dataset. Instead, interactions between QTN only occur under specific combinations of environment and genetic background. Thus, what might appear to be a QTN:QTN interaction in one background and environment becomes a more complex QTN:QTN:environment:background interaction when we consider the entire dataset as a whole. As a result, the phenotypic impact of a set of QTN alleles cannot be predicted from genotype alone. Our results instead demonstrate that the effects of QTN and their interactions are inextricably linked both to genetic background and to environmental variation.

## Author Summary

Phenotypic variation among individuals is caused by naturally occurring genetic differences, or alleles. The relationship between an allele and the phenotype is extremely complex; for example, the effect of an allele often depends upon both the environment and the individual's genetic background. To better understand these complex relationships, we examined the effects of four quantitative trait nucleotides (QTN) in three genes that cause variation in sporulation efficiency between vineyard and oak tree strains of yeast. We measured the effects of the QTN while varying both the genetic makeup of the strains and their growth environments. We found that the effects of each of the four QTN alleles depended upon the genotypes at the other QTN, the growth environment, and whether the strain carried the oak or vineyard parent genome. There were no simple rules that describe the effects of the alleles across all environments; instead, detailed models were needed to account for environmental and genetic variation in order to predict the effects of alleles in specific individuals.

## Introduction

As we identify more genetic loci that underlie complex traits, the challenge remains to understand and predict the effects of the causal genetic variants upon individuals' phenotypes. The relationship between genotype and phenotype is rarely simple. The effect of an allele often depends upon the environment, resulting in gene-environment interactions (GxE). GxE is a well-documented occurrence in many species, including humans [1]–[5]. Gene-gene interactions also take place that render the effect of one locus dependent upon the genotype at another locus. Genetic interactions can occur between characterized loci (epistasis) [6], [7], or between one known locus and other unknown loci (genetic background effects) [8]. If individuals vary in their environmental exposure and genetic makeup, as they almost always do in nature, then GxE and genetic interactions will create differences in the effects of alleles among individuals.

Therefore, to understand allelic effects, we must also understand the scope and prevalence of genetic and environmental interactions. However, standard approaches for the identification of causative loci, such as association analysis and linkage mapping [9], measure the average effects of alleles in populations. Without very large sample sizes, population averages cannot account for potential individual-to-individual variation created by complex interactions [10]. Some study of interactions on an individual-to-individual basis has occurred through the use of near isogenic lines [11], but there are still few examples that illustrate the impact of interactions from one individual to the next at the resolution of single-nucleotides [7], [12].

To better understand the effects of GxE and genetic interactions at the resolution of single nucleotides, we took advantage of four naturally occurring quantitative trait nucleotides (QTN) known to cause variation in yeast sporulation efficiency [13], [14]. We engineered allele replacement strains that carry all possible combinations of these QTN in two genetic backgrounds, and we then systematically measured the phenotypes of these strains in eight environments. Our results provide a detailed picture of how segregating QTN, environmental variation, and genetic background all combine to shape variation in a quantitative trait through complex relationships.

## Results

Our phenotype of interest, yeast sporulation, is a cell fate decision executed by diploid yeast cells in response to a shift from fermentative to respiratory conditions [15]. Yeast cells switch to primarily aerobic respiration when faced with only a non-fermentable carbon source. When this environmental change is accompanied by a reduction in a critical nutrient such as nitrogen, a fraction of yeast cells in a culture will initiate meiosis and enclose the meiotic products in a protective spore wall.

Our QTN all affect the proportion of cells in a culture that initiate meiosis (the sporulation efficiency) after a shift from glucose (fermentable) to acetate (non-fermentable) media. The QTN include a coding polymorphism in *RSF1* (a positive regulator of respiration) [16], both coding and non-coding polymorphisms in *IME1* (the master regulator of sporulation) [17], and a non-coding polymorphism in *RME1* (a direct repressor of *IME1*) [18], [19]. Each of these genes encodes a transcription factor.

Each QTN has two alleles: a reference allele found in the wild oak tree isolate YPS606, and an allele that reduces sporulation efficiency from the vineyard isolate UCD2120 [14]. (In the rest of this article, we denote the QTN with the labels: *rsf1*, *rme1*, *ime_coding*, and *ime_nc.*) Both the patterns of phenotypic variation in sporulation efficiency and the sequence variation of the causal genes indicate that sporulation efficiency is subject to purifying selection in oak strains and disruptive selection in vineyard strains [14], [20]. The change in phenotype caused by the QTN therefore represents genotype-phenotype variation that has occurred due to a shift in selective pressures between two habitats.

To broadly test for GxE effects, we first measured the sporulation efficiency of each parent strain genetic background (designated oak and vineyard) carrying two QTN genotype combinations: either all the QTN alleles of the oak parent, or all the QTN alleles of the vineyard parent. We generated environmental variation by growing the strains in eight different fermentative media conditions (Table 1) prior to the induction of sporulation in acetate (see Methods). In all eight environments and across both genetic backgrounds, the oak QTN alleles collectively increase sporulation efficiency, and the vineyard QTN alleles collectively decrease sporulation efficiency (Table 2). However, the environments vary with respect to the proportion of the phenotype that the QTN explain (Table 3). For example, in grape juice, we can explain 99% of the phenotypic difference between the parents by placing the vineyard QTN alleles into the oak background. However, in raffinose, the same allele replacement explains only 55% of the parental difference. The phenotypic difference explained by the QTN also depends on the genetic background. For example, placing the oak QTN alleles into the vineyard background explains 90% of the difference between the parent strains in raffinose, but we explain only 55% of the difference between the parents if we conduct the reciprocal experiment that places the vineyard QTN alleles into the oak background. Because the phenotypic difference created by the QTN varies across both the environments and genetic backgrounds, our results imply genetic interactions among the QTN, the environmental treatments, and uncharacterized loci in the two parent genetic backgrounds.

**Table 1:** Environments used in these experiments.

		Nutrient Concentrations	
Name	[Yeast Extract]	[Peptone]	[Carbon Source]
Glucose	1%	2%	2% Glucose
Fructose	1%	2%	2% Fructose
Sucrose	1%	2%	2% Sucrose
Maltose	1%	2%	2% Maltose
Raffinose	1%	2%	2% Raffinose
Grape Juice	NA	NA	NA
Exudate	0.10%	0.15%	1% Sucrose, 0.5% Glucose, 0.5% Fructose
Galactose	1%	2%	2% Galactose

Percentages are weight*volume^−1^.

**Table 2:** The effects and standard deviations, in each environment, of strains carrying all the QTN alleles from one parent.

Genetic Background:	Oak	Oak	Vineyard	Vineyard
QTN alleles from:	Oak	Vineyard	Oak	Vineyard
Glucose	99.3±0.2	17.5±3.4	74.6±2.5	4.1±0.5
Fructose	99.7±0.2	11.6±1.7	74.8±1.5	1.2±0.2
Sucrose	99.1±0.3	13.6±1.7	78.8±4.3	2.4±0.6
Maltose	99.2±0.3	16.7±2.0	80.1±2.1	0.8±0.1
Raffinose	97.5±0.2	48.2±6.2	88.0±1.8	5.0±0.1
Grape Juice	56.2±2.6	0.5±0.3	27.0±3.4	0.1±0.03
Exudate	79.0±0.7	4.1±1.1	44.9±1.8	0.3±0.1
Galactose	90.6±0.3	16.7±3.0	82.4±0.8	6.0±0.5

**Table 3:** Proportion of the difference between the parents accounted for by the QTN.

Environment	Oak Background	Vineyard Background
Glucose	0.86	0.75
Fructose	0.89	0.75
Sucrose	0.89	0.80
Maltose	0.84	0.81
Raffinose	0.55	0.90
Grape Juice	>0.99	0.48
Exudate	0.96	0.57
Galactose	0.89	0.91

To further investigate the extent of these interactions, we measured the phenotypes of strains with all 16 possible QTN genotype combinations in both genetic backgrounds (32 total strains). We calculated a correlation matrix of the eight environments from their effects on the phenotype rank-order of the 32 strains so that we can broadly compare the QTN effects across environmental treatments and genetic backgrounds (Table 4). Because the QTN alleles always act in the same direction regardless of condition, all the environments were positively correlated (Spearman's *ρ* = 0.69 to 0.99). The differences in correlations therefore reflect changes in the rank order (and therefore relative magnitude) of QTN effects.

**Table 4:** Matrix of Spearman's ρ across all environments.

	Glucose	Fructose	Sucrose	Maltose	Raffinose	Grape juice	Exudate	Galactose
Glucose	1.000	0.992	0.990	0.968	0.945	0.897	0.856	0.852
Fructose	0.992	1.000	0.995	0.955	0.922	0.870	0.826	0.865
Sucrose	0.990	0.995	1.000	0.945	0.917	0.870	0.834	0.888
Maltose	0.968	0.955	0.945	1.000	0.974	0.930	0.898	0.729
Raffinose	0.945	0.922	0.917	0.974	1.000	0.956	0.923	0.722
Grape Juice	0.897	0.870	0.870	0.930	0.956	1.000	0.973	0.708
Exudate	0.856	0.826	0.834	0.898	0.923	0.973	1.000	0.687
Galactose	0.852	0.865	0.888	0.729	0.722	0.708	0.687	1.000

**Figure 1:**
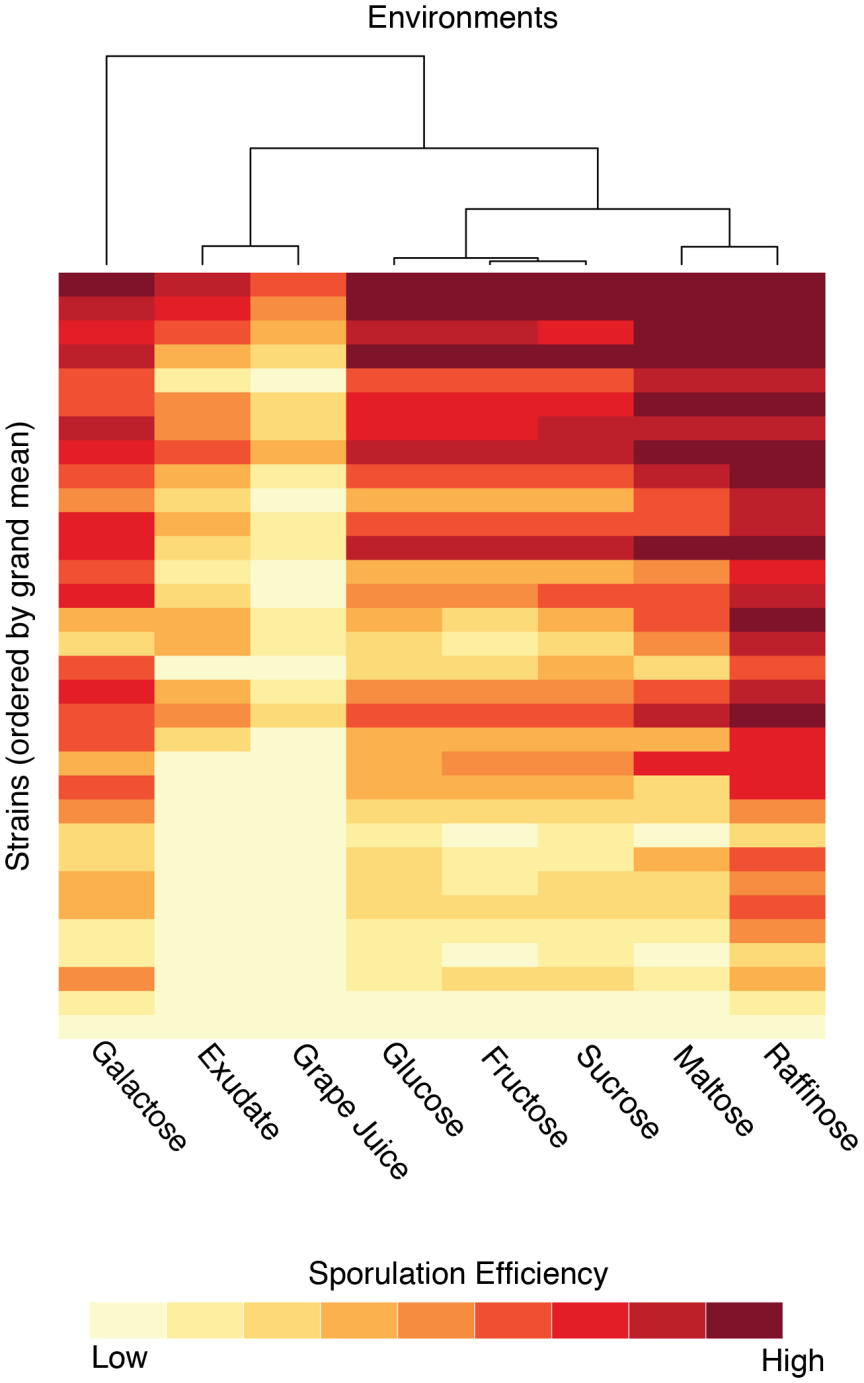
Sporulation efficiencies of the strains clustered by environment. The dendrogram is constructed from Spearman's *rho* between environments. The heatmap shows the sporulation efficiencies of the 32 strains in the panel, which consist of all sixteen combinations of the four QTN in both the oak and vineyard backgrounds. The 32 strains are ordered according to their grand mean values across all environments.

We used hierarchical clustering to construct a dendrogram that reflects the correlations between environments (Figure 1). Sucrose, fructose, and glucose are the most highly correlated environments (Spearman's *ρ*>0.99 for all pair wise comparisons) and cluster closely. We did not detect significant differences between these three environments in either genetic background. Their values were therefore pooled and averaged as “glucose-like” (YGlu) for all subsequent analyses.

**Figure 2:**
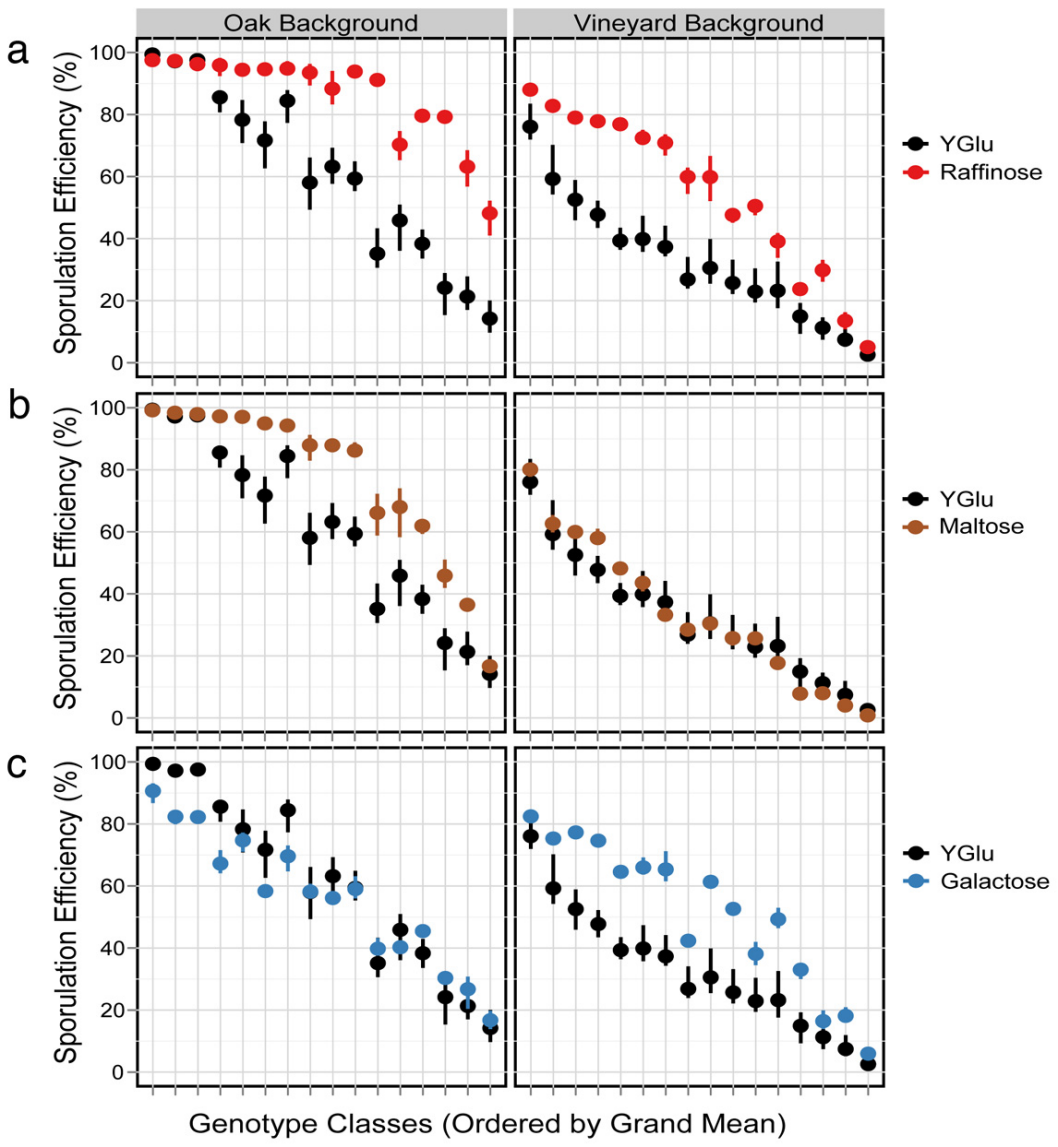
Effect of rich media environments (1% yeast extract, 2% peptone) on sporulation efficiency. Sporulation efficiencies of the strains are split by genetic background. (A) Raffinose. (B) Maltose. (C) Galactose. On the x-axis, the 16 QTN genotype combinations are ordered by their grand mean values across all environments and both genetic backgrounds. Points denote the mean of each strain, and vertical bars denote the full range of values. N = 3 for all environments except YGlu, where N = 9.

Maltose and raffinose cluster separately from YGlu and are slightly less correlated with glucose (*ρ* = 0.93, 0.96, respectively). Both the oak and vineyard genetic backgrounds sporulate more efficiently in raffinose than in YGlu. (Figure 2A) The effect of maltose, however, depends upon the genetic background (Figure 2B). Sporulation efficiency of the vineyard background is similar in maltose and YGlu, but the oak background sporulates more efficiently in maltose. Therefore, there is an interaction between the genetic background and maltose.

Galactose also shows a background:environment interaction. The oak background sporulates similarly in galactose and YGlu, but the vineyard background sporulates more efficiently in galactose (Figure 2C). In Figure 1, galactose clusters distinctly from all other environments. When we run the clustering algorithm separately for each genetic background, this separation disappears (Figures S1, S2). Therefore, the disparity of galactose relative to the other environments appears to result from the background:environment interaction.

**Figure 3:**
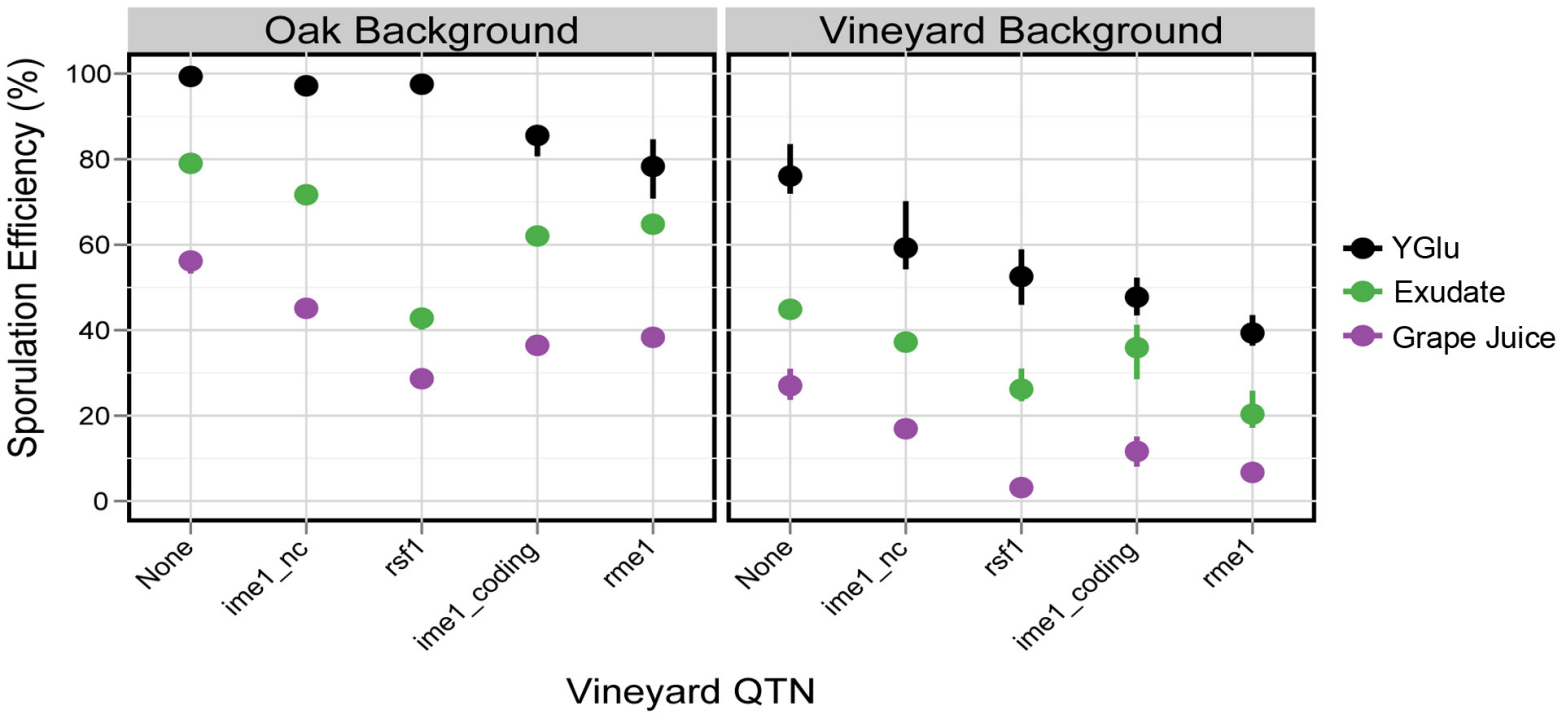
QTN effects in exudate and grape juice. In each genetic background, the strain carrying the oak alleles (None) is plotted along with strains carrying single vineyard QTN alleles. In the oak background, *rsf1* has a small effect in YGlu but the largest effect of any QTN in exudate and grape juice. In the vineyard background, the effect of *rsf1* is similar across all three environments. Points denote the mean values of each strain, and vertical bars denote the range. N = 3 for exudate and grape juice, and N = 9 for YGlu.

Synthetic oak exudate and grape juice also cluster distinctly from the other environments, but these two conditions are highly correlated with each other (*ρ* = 0.97). The sporulation efficiency of both genetic backgrounds tends to be lower in exudate and grape juice than in the other environments (Figure 1, Tables S1, S2). There are also QTN:environment interactions that occur in both exudate and grape juice relative to YGlu. For example, in the oak background, the *rsf1* vineyard allele has a much larger effect in exudate and grape juice than it does in YGlu (Figure 3). To quantify this difference in QTN effect, we constructed a linear model of sporulation efficiency in the oak background that incorporates the main effects of single QTN, the effects of the environments, and the QTN:environment interactions (see Methods). In this model, the differential effect of *rsf1* is manifested as a QTN:environment interaction in exudate and grape juice relative to YGlu (exudate: effect = −34±2%, t-test, *P*<2e-16; grape juice: effect = −25±2%, t-test *P*<2e-16; all errors reported in the text are the standard errors of coefficient estimates). The effect of *rsf1* is the largest of any single QTN in both grape juice and exudate (Tukey's HSD, maximum adjusted *P* = 0.002). However, in YGlu the *rsf1* QTN does not even have a significant main effect in the oak background (effect = −1.8±1%, t-test *P* = 0.08).

The effect of *rsf1* in the vineyard background reveals a different story. In the vineyard background, the *rsf1:*environment interaction is not significant in exudate or grape juice (exudate: effect = 4.8±2.8%, t-test *P* = 0.1; grape juice: effect = −0.3±0.03%, t-test, *P* = 0.92). Instead, *rsf1* has a large main effect in YGlu as well as exudate and grape juice (Figure 3). Therefore, the effect of *rsf1* can be best explained as an environment:*rsf1*:background interaction that reduces the effect of *rsf1* in YGlu relative to exudate and grape juice, but only in the oak strain background.

How do the effects of these environment and background interactions compare with the role played by QTN:QTN interactions? We previously demonstrated significant QTN:QTN epistasis in the oak background and the glucose environment [14]. In that context, epistasis appears to play a large role in shaping phenotypic variation. However, the differences in *rsf1's* effect across backgrounds and environments imply that the QTN:QTN interactions might occur only in certain environments or backgrounds. We therefore tested for all possible QTN:QTN interactions across all eight environments and both genetic backgrounds.

**Figure 4:**
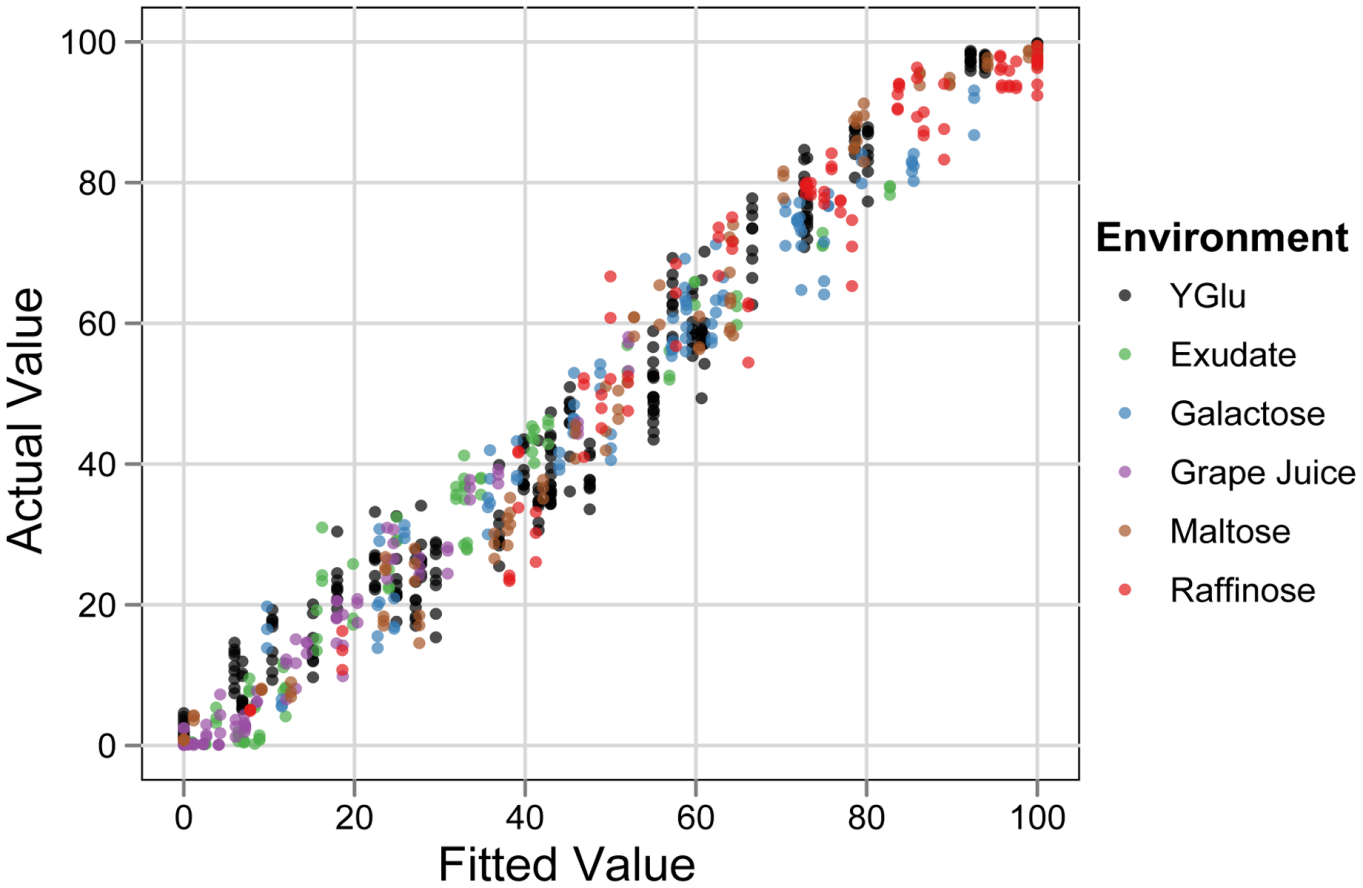
Linear model of sporulation efficiency. Actual sporulation efficiency of strain replicates plotted as a function of the values fitted from the global model, which uses three-way interactions to account for QTN genotypes as well as genetic background and environment. Fitted values were forced to fall between the range of 0 and 100%. The average deviation of all points from their fitted values is 4.6%.

To do so, we modeled variation in sporulation efficiency in a standard linear framework using all phenotypic measurements across QTN genotypes, genetic backgrounds, and environments (see Methods). A completely saturated model that incorporates all possible effects and interactions between environment, background, and QTN has an adjusted *R^2^* of 0.99. All of the parameters in the model are controlled variables, so this *R^2^* indicates that 1% of the variation in our experiment is due to experimental error. We then constructed a reduced model that explains most of the variation, but with fewer parameters and only two and three-way interactions (Figure 4, adjusted *R^2^* = 0.963, see Methods). This model (the global model) captures the predominant interactions in the data (Table S3). For example, it contains a significant positive interaction term between galactose and the vineyard background (effect = 25.6±2.4%, t-test *P*<2e-16). This term is expected given the higher sporulation efficiencies we observe in the vineyard background in galactose (Figure 2C). There are also significant interactions between *rsf1* and both exudate (effect = −28±2.8%, t-test *P*<2e-16) and grape juice (effect = −13.8±2.8%, t-test *P* = 9e-7), which are expected due to the larger effect of *rsf1* in these two conditions (Figure 3).

The most striking result from the global model is the lack of two-way QTN:QTN interactions. Three QTN:QTN interaction terms were left in the model after stepwise regression (Table S3). Only one of these, a negative interaction between *rsf1* and *ime1_coding* (effect = −7.6±1.8%, *P* = 3.9e-5), passed either Bonferonni correction or permutation testing. This result stands in contrast to what we observe within a single condition. In line with our previous data in glucose [14], we find abundant QTN:QTN interactions when YGlu is modeled alone (Table S4). For example, the *rme1*:*ime1_coding* interaction is large (effect = −29.4±2.3%, t-test *P*<2e-16). However, when all the environments and both backgrounds are analyzed together in the global model, the same *rme1*:*ime1_coding* interaction is small and only marginally significant (effect = −3.9±1.6%, t-test *P* = 0.02).

**Figure 5:**
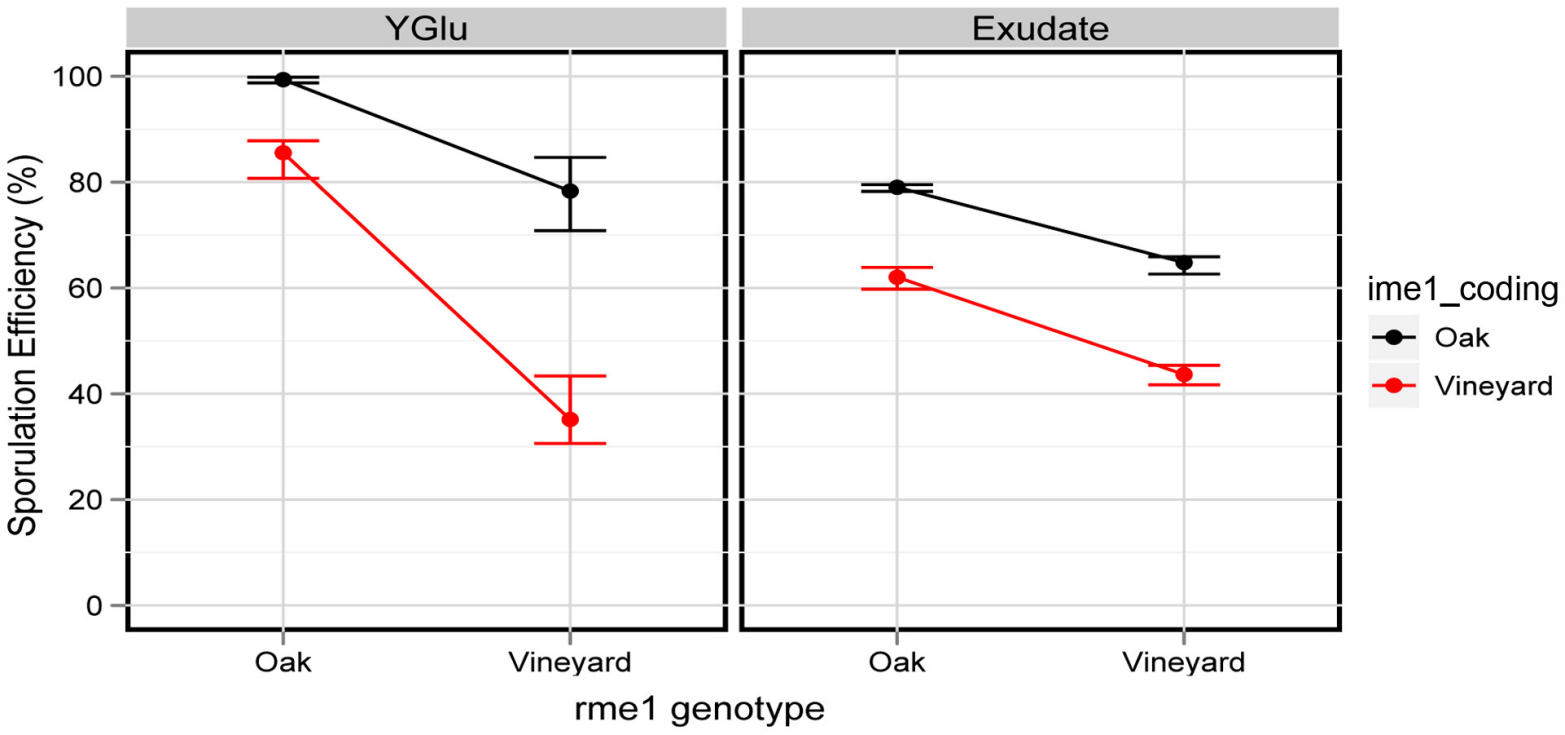
The *rme1:ime1_coding* interaction is environment-dependent in the oak background. The interaction plots show the *rme1* genotype on the x-axis. The *ime1_coding* genotype is signified by the black (oak allele) and red (vineyard allele) lines. The left panel shows the phenotypes of allelic combinations in YGlu, and the right panel shows the same allelic combinations in exudate. The vineyard polymorphisms have a synergistic effect in YGlu, as evidenced by the change in slope between the two lines. The polymorphisms have independent effects in exudate, as evidenced by parallel lines. Points denote the mean of each strain and error bars denote the range.

**Figure 6:**
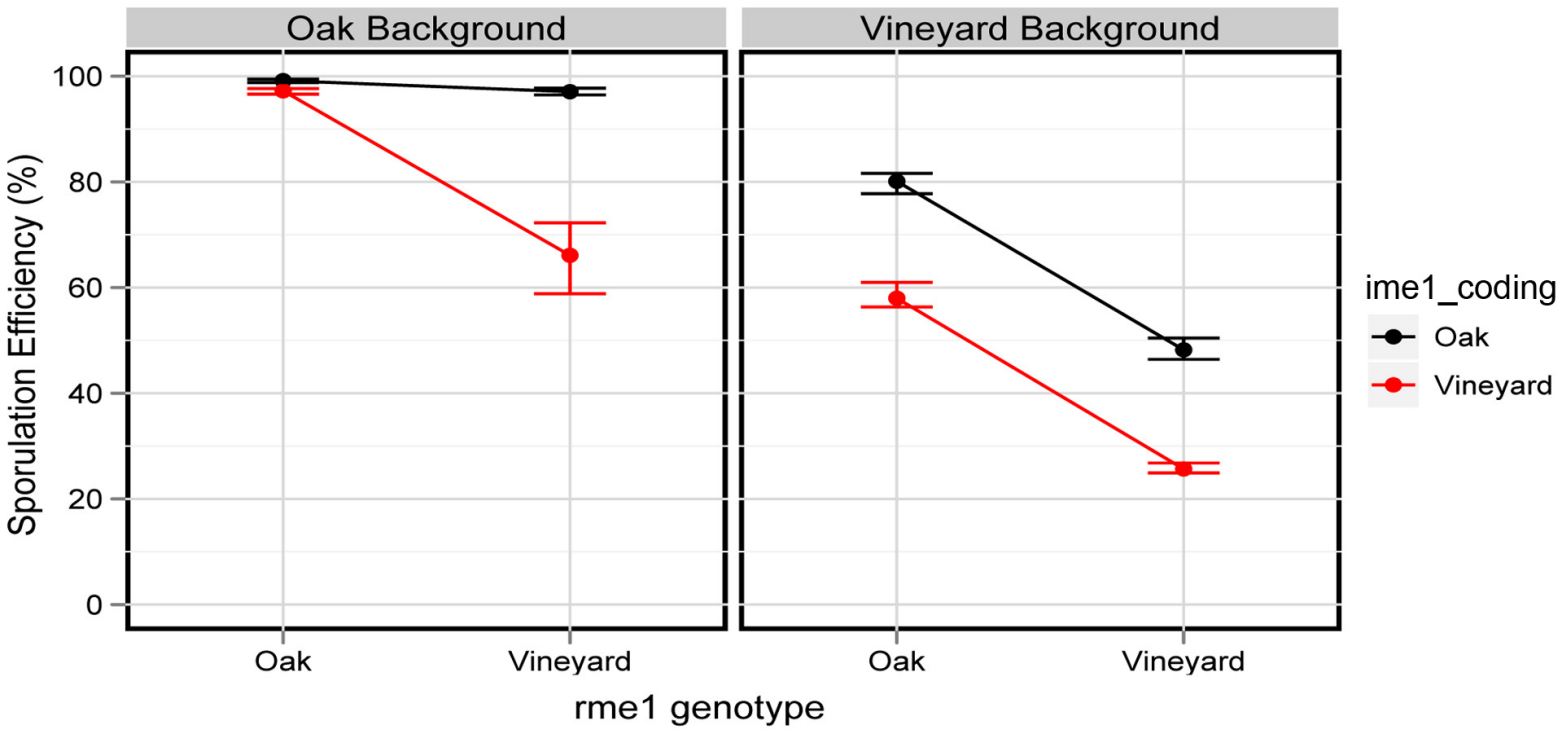
The *rme1:ime1_coding* interaction is background-dependent. Interaction plot of the allelic set in Maltose. The left panel shows the allelic combinations in the oak background, and the right panel shows the vineyard background. An interaction occurs in the oak background, as evidenced by the change in slope between the two lines. This interaction does not occur in the vineyard background, as evidenced by parallel lines. Points denote the mean of each strain and error bars denote the range.

In the place of QTN:QTN interactions, the global model contains several significant three-way QTN:QTN:environment and QTN:QTN:background interactions. This suggests that significant QTN:QTN interactions cause variation in sporulation efficiency, but the interactions only occur in particular environments and genetic backgrounds. To examine this possibility further, we modeled each environment-background combination separately and observed that QTN:QTN interactions varied widely. For example, the *rme1:ime1_coding* interaction that is strong in YGlu in the oak strain is marginal in exudate (Figure 5, YGlu effect = −29.4±2.3%, t-test *P*<2e-16 ; exudate effect = −4.2±2.1% , t-test *P* = 0.052). This interaction is present in maltose (effect = −29±3.9%, t-test *P* = 1.6e-8), but not in the vineyard background (Figure 6, effect = 0.00±2.1%, t-test *P* = 0.23). Taken together, these results show that the vineyard alleles of *rme1* and *ime1_coding* act synergistically in the oak strain and specifically in YGlu and maltose, as the combination of two vineyard QTN produces a larger change in phenotype than could be expected from their individual effects. However, in exudate, or in the vineyard background, the effects of these same QTN alleles remain independent. Therefore, the synergistic interaction between the vineyard alleles is not intrinsic to the alleles themselves, but instead depends upon the specific context of the environment and genetic background.

**Figure 7:**
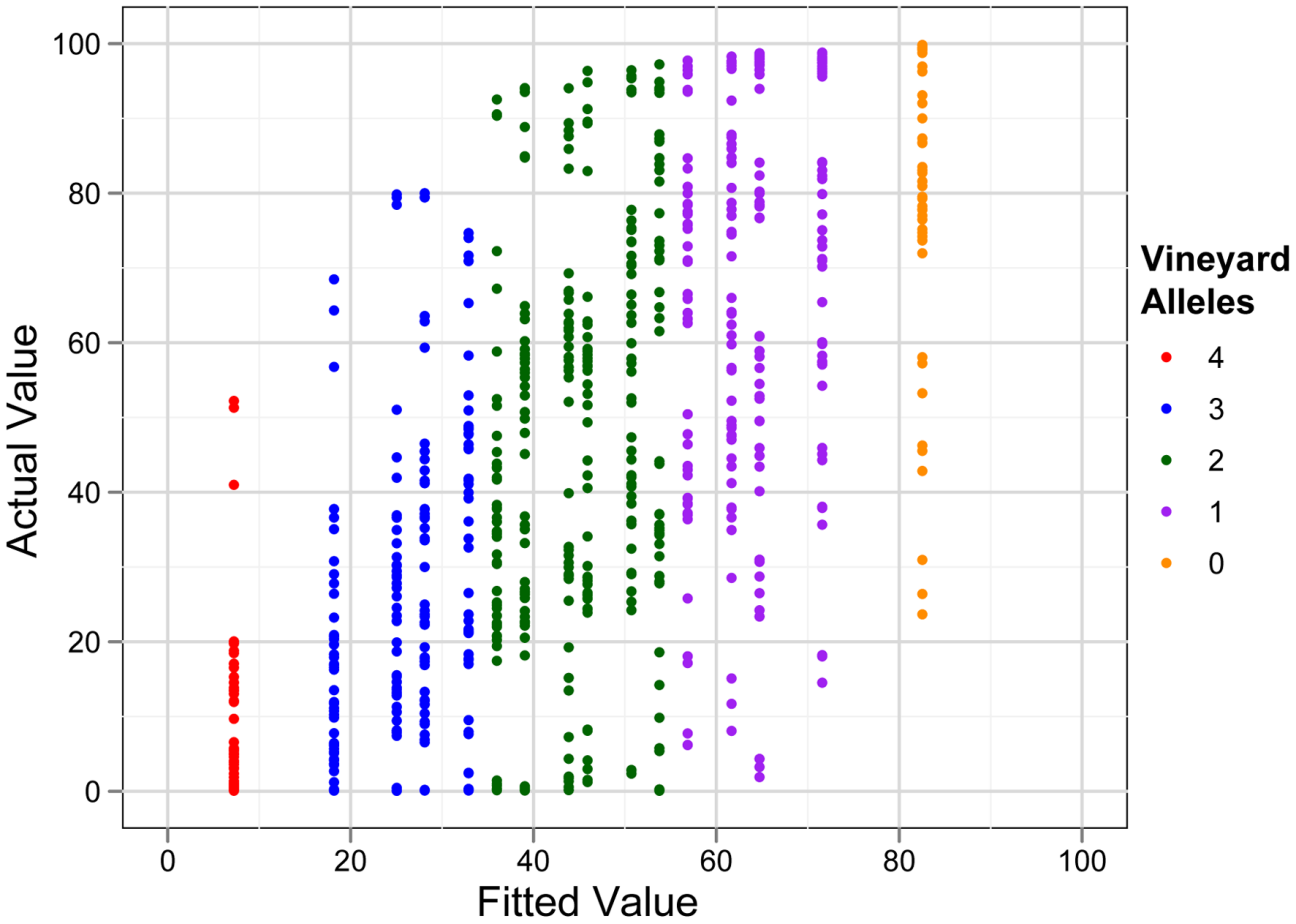
QTN only model of sporulation efficiency. Actual versus fitted values of a statistical model when environment and genetic background are uncontrolled. The fit is poorer when environment and background are ignored (compare to Figure 4). The model fit is better for strains with all vineyard QTN alleles (red points) than for strains with mixtures of oak and vineyard alleles.

Our measurements of sporulation efficiency therefore indicate that QTN:QTN interactions are not widespread, but QTN:QTN:environment and QTN:QTN:background interactions are common. In a linear model that ignores genetic background and environment, no interactions between the QTN are significant (adjusted *R^2^* = 0.4). This QTN-only model correctly identifies that individuals with all vineyard alleles tend to sporulate poorly, but it does not provide the ability to accurately predict the phenotypes of individuals with intermediate genotypes (Figure 7). Ultimately, the effects of the QTN and their interactions are shaped by the environmental and genomic context in which they occur. Knowledge of the environment and genetic background is therefore crucial to accurately predict the effects of QTN across individuals (compare Figure 4 to Figure 7).

## Discussion

In this set of experiments, we measured sporulation efficiency in a variety of isogenic strains that differed with respect to QTN genotypes, genetic background, and growth environment. Overall, our results show that a complex set of genotype:environment:background interactions shape variation in sporulation efficiency. Our results also shed light on the general effects of environment on sporulation efficiency in the context of natural variation. We found that carbon sources with similar effects on yeast catabolite repression tended to have similar effects on sporulation efficiency. For example, glucose and fructose both cause strong catabolite repression in yeast [21], and their effects on sporulation efficiency are highly correlated (Table 4). Sucrose, a disaccharide composed of glucose and fructose, is likewise highly correlated with glucose. Raffinose and galactose, which cause weaker catabolite repression [22], cluster less closely with glucose. One surprising result is the GxE we observed in maltose relative to glucose (Figure 2B). Since maltose is composed of two glucose molecules, one might expect the effect of maltose to be as similar to glucose as that of sucrose or fructose. One possible explanation for the GxE in maltose arises from the fact that maltose catabolism genes commonly display copy number variation among yeast isolates [23]–[25]. We observed a slow growth phenotype of the oak strain in maltose and mapped this phenotype to the MAL1 multigene locus (K. Lorenz and B. Cohen, unpublished results). We suspect that this locus is responsible for the maltose:background interaction we observe for sporulation efficiency, and it may also modulate the QTN effects and QTN:QTN interactions in maltose, but confirmation of this hypothesis awaits the cloning of the causative polymorphism.

Exudate and grape juice produce lower sporulation efficiencies than the other environments. This result occurs in spite of the fact that exudate is composed of exactly the same ingredients as YGlu, but with reduced concentrations of peptone and yeast extract. This reduction of nutrient concentrations not only reduces sporulation efficiency in both genetic backgrounds, but it also alters the effect of *rsf1* in the oak background relative to the other QTN (Figure 3). The fact that exudate consists of the same ingredients as YGlu but produces different effects on sporulation efficiency suggests that QTN effects are shaped not only by nutrient type, but also by nutrient concentrations. Drops in nitrogen concentration are well-known to strengthen the signal to sporulate, so the difference in peptone concentration between exudate and rich media may explain some the differences in sporulation efficiency through nitrogen sensing.

Across multiple environments, the unknown polymorphisms in the genetic background not only interact with the environment but also alter the effects of the known QTN. The known QTN used in this study were mapped in glucose and explain ∼90% of the segregating variation in that condition [14]. The interactions we observe here suggest that the remaining unmapped loci may have stronger effects (and be easier to map) in non-YGlu environments. For example, the known QTN only explain half of the phenotypic difference in the vineyard background in grape juice (Table 3). Presumably, the remaining unknown polymorphisms that regulate sporulation efficiency have larger effects in this environment-background combination than they do in YGlu. An attractive experiment to identify new QTN governing sporulation efficiency would therefore be to map the phenotype in grape juice using a cross of the original oak parent with a new version of the vineyard parent strain that is fixed for all four known oak QTN. It is possible, however, that the new polymorphisms uncovered by this experiment would not reside in the sporulation pathway *per se*, but would instead be metabolic factors specific to grape juice catabolism.

Despite the fluctuations in QTN effects across environments and backgrounds, the direction of QTN effects remain consistent. Vineyard alleles always decrease sporulation efficiency relative to oak alleles. Without accounting for changes in the environment or differences in genetic background, we can therefore safely predict that a strain with all four vineyard alleles will sporulate poorly relative to a strain carrying all oak alleles. However, because the effect magnitudes of the QTN change across environments and backgrounds, we cannot predict the sporulation efficiency of intermediate allelic combinations (Figure 7). This case reminds us of the situation unfolding in human association studies, where it appears that high-risk individuals can be identified as carriers of collections of disease associated polymorphisms, even though it is more difficult to predict the actual phenotypic outcome of a particular individual with intermediate sets of alleles [26]. In this case of yeast sporulation efficiency, complexity occurs because the relative importance of particular alleles and their interactions are not constant across individuals, but instead vary with the individuals' genetic background and environment.

If context dependencies on allelic effects are common, how can we achieve better predictive power when environment and background are unknown? Environment and genetic background presumably influence the phenotype just as all genetic changes must: through effects on cell physiology. It might be possible to account for the physiological effects of environment and background using a biomarker or physiological indicator that is correlated with, but upstream of, the phenotype of interest. Biochemical markers are used in medicine to inform calculations of disease risk and diagnosis [27]. Inclusion of a physiological marker into the genetic model may condition the model to unknown parameters and therefore increase the accuracy of genotype-phenotype predictions.

Although such a model could improve predictive power, it still does not increase our understanding of how various physiological forces in the cell combine to quantitatively alter phenotype. Perhaps improved understanding could arise from interpreting QTN effects through a framework rooted in cell biology and biochemistry, rather than through an abstract linear model. Biochemical and gene regulatory pathways have long been theorized to naturally generate non-linear effects through the basic thermodynamic properties of proteins and DNA [28], [29]. We have modeled sporulation efficiency in glucose through a thermodynamic framework, and this method shows promise in revealing the molecular basis of genetic interactions [30]. However, thermodynamic modeling requires detailed knowledge of molecular mechanism of the proteins involved, and this information is not available for most traits. Also, the challenge of applying this approach to multiple environments is nontrivial [31].

A more traditional method to deal with statistical interactions is to eliminate them through data transformations. We experimented with a number of scale transformations for our dataset, but found that the best transformation for reducing the complexity of the interaction terms varied from one environment:background combination to the next. Furthermore, data transformations that reduced the number of interaction terms sometimes had undesirable effects, such as increasing the dependence of the variance upon the mean. More importantly, scale transformations that worked well on some subsets of the data still required numerous interaction terms to provide a global model. None of the data transformations we tried improved the three-way interaction fit obtained on the natural scale (Figure S3). Although data transformations may be appropriate to obtain simpler predictive models in single background:environment combinations, they do not account for the non-linear dynamics that create complexity across conditions and backgrounds.

Regardless of the approach taken in the future, our results clearly show that the genetic architecture of sporulation efficiency is environment-dependent. QTN effects cannot be understood without taking into account contextual factors such as the environment's influence on cell physiology. We expect that quantitative biochemical measurements will be required to illuminate what is happening inside the cell and bridge the missing link between genotype and phenotype.

## Methods

### Experimental Design

Each of the 32 strains were grown for 15 hours in growth media (except for grape juice, in which we instead grew the yeast for 54 hours). After the growth period, we diluted each culture 1∶50 into 1% potassium acetate to induce sporulation. We tested three replicates of each QTN genotype - environment - genetic background combination. One exception is the strain carrying only the *ime_nc* vineyard QTN allele in the vineyard background grown in sucrose, for which there were only two measurements due to a sample failure. The experimental design is balanced such that the genotype frequencies of the four QTN do not vary across environments or backgrounds, so any significant interactions between QTN reflect physiological effects rather than differences in allele frequency [32]. Sporulation efficiency was calculated by flow cytometry on samples of 15,000 cells per replicate using methods we have described elsewhere [20]. The raw data of sporulation efficiencies for each replicate is available as a supplementary data file (Dataset S1).

Each of the eight environmental treatments was composed of a different growth medium prior to the induction of sporulation in acetate (Table 1). Six of the environments consisted of rich yeast media (1% yeast extract, 2% peptone) supplemented with 2% of a sugar or polysaccharide: glucose, fructose, sucrose, maltose, galactose, or raffinose. The other two environments were synthetic oak exudate and chardonnay grape juice. Synthetic oak exudate is composed of the same nutrients as rich media, but contains yeast extract and peptone at ten-fold reduced concentrations (Table 1). Exudate also contains a mixture of fructose, sucrose, and glucose at a total concentration of 2% [33]. After each environmental treatment, sporulation was induced for 30 hours in 1% potassium acetate, which provides a non-fermentable carbon source but no source of nitrogen.

### Strain Construction

First, we created allele replacement strains in each parental background that carry single QTN alleles from the opposite parent [34]. These strains were created by backcrosses of initial haploid *ura3−* allele replacement transformants with their prototrophic diploid parents. *Ura3+* progeny from the backcross of each allele replacement were then intercrossed to generate strains carrying multiple QTN alleles from the opposite parent. Each cross was performed in triplicate. We confirmed after each cross that the QTN co-segregated with variation in sporulation efficiency in glucose, and we also ensured that the phenotypes resulting from replicate crosses were identical. This assured us that no new mutations governing sporulation efficiency had arisen elsewhere in the genome during the crossing scheme. Once a strain with the desired QTN alleles from the opposite parent was created, this strain was backcrossed once more to its original wild type parent strain. Individual homothallic diploid progeny from this final cross were isolated and genotyped until we obtained three replicates of every possible QTN allele combination. Genotyping was based on the restriction digest of PCR amplicons [14]. The selected strains were arrayed in a 96-well plate such that all the strains from both genetic backgrounds can be assayed in a single block.

### Non-Parametric Cluster Analysis

We generated a matrix of the Spearman rank correlations of the means of each of the 32 strains across each environment. A distance matrix was then defined as 1−*ρ*, where *ρ* is the matrix of pair wise Spearman rank correlations. We carried out hierarchal cluster analysis with the complete linkage clustering method as implemented in the *hclust* function in the statistical package R. We also split the data by genetic background, then calculated rank correlations and clustered separately for the oak and vineyard genetic backgrounds.

### Linear Models

All statistical analyses were performed in R. In all linear models, the strain with all oak QTN alleles was treated as the intercept, so the additive effects represent the effect of a single vineyard QTN placed into a strain with oak QTN alleles at all other loci. We chose this reference point because the oak strain probably best resembles the genotype of the common ancestor of the two parent strains [14], [35]. To compare the effects of QTN:QTN interactions in single environment-background combinations, we created linear models of QTN effects including all possible interaction terms within each condition, and significant coefficients were calculated by t-tests of the coefficient's estimated effect versus its standard error. All interaction terms reported in the text are significant by Bonferonni correction (*P* = 0.05/N, where N is the number of coefficients in the model).

To analyze QTN effects across all environments and both backgrounds, we constructed a linear model in which the oak genetic background, oak QTN alleles, and the glucose environment are treated as intercepts. Therefore, coefficients in the model represent the effects of the vineyard genetic background, vineyard QTN alleles, and non-glucose environments. The simplest additive model therefore takes the following form:
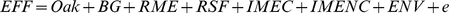
Where *EFF* is sporulation efficiency, *Oak* is the oak strain phenotype in glucose (the y-intercept), *BG* is the effect of genetic background, *RME*, *RSF*, *IMEC*, and *IMENC* are the effects of the vineyard QTN alleles, *ENV* is the effect of non-glucose environments, and *e* represents the error across the multiple replicates of each combination of strain and environmental treatment. Sucrose and fructose were not significantly different from glucose, so these three conditions were pooled into a single treatment. We found that models with increasing levels of interaction terms were often significant, but very little improvement to the fit or explanatory power of the model was gained by adding four-way interactions (Table S5). We therefore limited our analysis to models with three-way interaction terms to reduce saturation without much sacrifice of explanatory power. To select a specific model with a subset of the three-way terms, we used stepwise regression as implemented in the *stepAIC* function in R. We then took the output from stepwise regression and manually removed terms from the model if their treatment contrast *P*-values did not pass a model-wide Bonferonni correction. Table S3 displays the coefficients in our final model and the *P*-values of each coefficient. The significance of the QTN:QTN interactions in this model were also tested by creating 10,000 null linear models from random permutations of the entire dataset. The critical P-value from these permutations was *P* = 0.016.

Probit and Logit transformations, which are common used for frequency data, provide good fits with fewer interaction terms in some individual conditions. However, we chose to model the data on the raw scale. The Probit and Logit transformations obtain a fit by weighting the explanatory power at extremely high and low values of sporulation efficiency at the expense of intermediate values (Figure S3). For example, under the Probit transformation, a difference in sporulation efficiency from 1% to 2% is as great in magnitude as a raw difference of 40% to 50%. No transformation eliminated interactions altogether, and transformations did not improve the overall fit of the model across multiple environments. The raw scale allows more intuitive interpretation of the model coefficients, and our reduced model performs well on values of sporulation efficiency between ∼5 and 95%. Some extreme values are fit below zero or above 100%. However, with one exception (a data point at 92%), all data points fitted to higher than 100% have actual values greater than 96%. All data points predicted to be below zero have actual values less than 5%. We therefore simply bounded all predicted values between 0% and 100%.

To model the specific QTN:environment interactions in exudate and grape juice, we conducted an analysis of variance on only the additive effects (no QTN:QTN interactions) of the four vineyard QTN separately in each genetic background. This model focused on the additive effects because the phenotypes of vineyard background strains carrying multiple vineyard QTL approach zero in a non-linear fashion (Tables S1, S2). The model took the form:
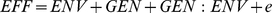
Where *EFF* is sporulation efficiency, *GEN* is the genotype across the four QTN alleles, *ENV* is the environment, and *e* is the error. To confirm significant differences in the rank order of QTN effects in different environments, we took the estimated QTN effects from an analysis of variance in each environment separately and computed Tukey's Honest Significant Difference to determine the rank-order of the QTN within each environment. The reported *P* value is the largest adjusted *P* value among all the possible comparisons between the effect of *rsf1* and the effects of other QTN.

## Supporting Information

Dataset S1 The sporulation efficiencies for each replicate.
(0.02 MB TXT)

Figure S1 Heatmap produced by clustering sporulation efficiencies in the oak background only.
(0.15 MB TIF)

Figure S2 Heatmap produced by clustering sporulation efficiencies in the vineyard background only.
(0.15 MB TIF)

Figure S3 Three-way interaction models of sporulation efficiency after scale transformations. Actual values for each strain replicate are denoted on the x-axis, and the predicted values are on the y-axis. (A) The raw linear scale. (B) Arcsine transformation. (C) Logit transformation. (D) Probit transformation. These scale transformations reduced the number of interaction terms in models of some single environments.
(0.21 MB TIF)

Table S1 Mean % sporulation efficiency for each QTN-background combination in each environment. O = oak genotype, V = vineyard genotype.
(0.03 MB XLS)

Table S2 Standard deviation of % sporulation efficiency for each QTN-background combination in each environment. O = oak genotype, V = vineyard genotype.
(0.03 MB XLS)

Table S3 Coefficients in our final model of sporulation efficiency. Coefficients with P values that pass bonferroni correction (P<0.00075) are labeled with an asterisk.
(0.03 MB XLS)

Table S4 Coefficients from a model of the oak background in Yglu.
(0.03 MB XLS)

Table S5 The residual degrees-of-freedom and adjusted r2 of linear models of sporulation efficiency with varying levels of interaction terms.
(0.02 MB XLS)
