# Supplementary figures and images for: Gene–Environment Interactions at Nucleotide Resolution
Source: PLoS Genet. 2010 Sep 30;6(9):e1001144. doi: 10.1371/journal.pgen.1001144 (PMC2947989; doi:10.1371/journal.pgen.1001144)

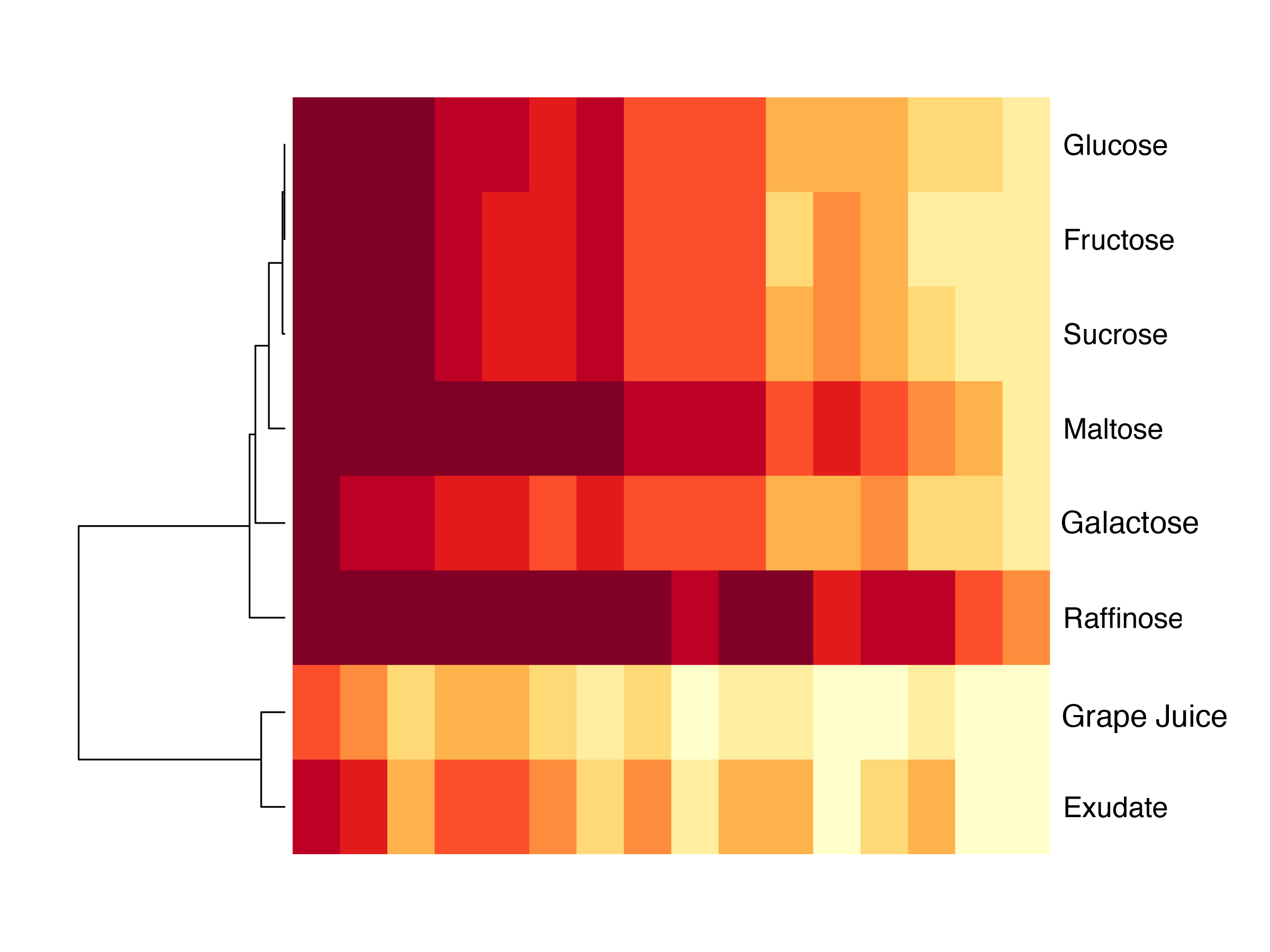

Supplement: Figure S1 — Heatmap produced by clustering sporulation efficiencies in the oak background only. (0.15 MB TIF) [file pgen.1001144.s002.tif]

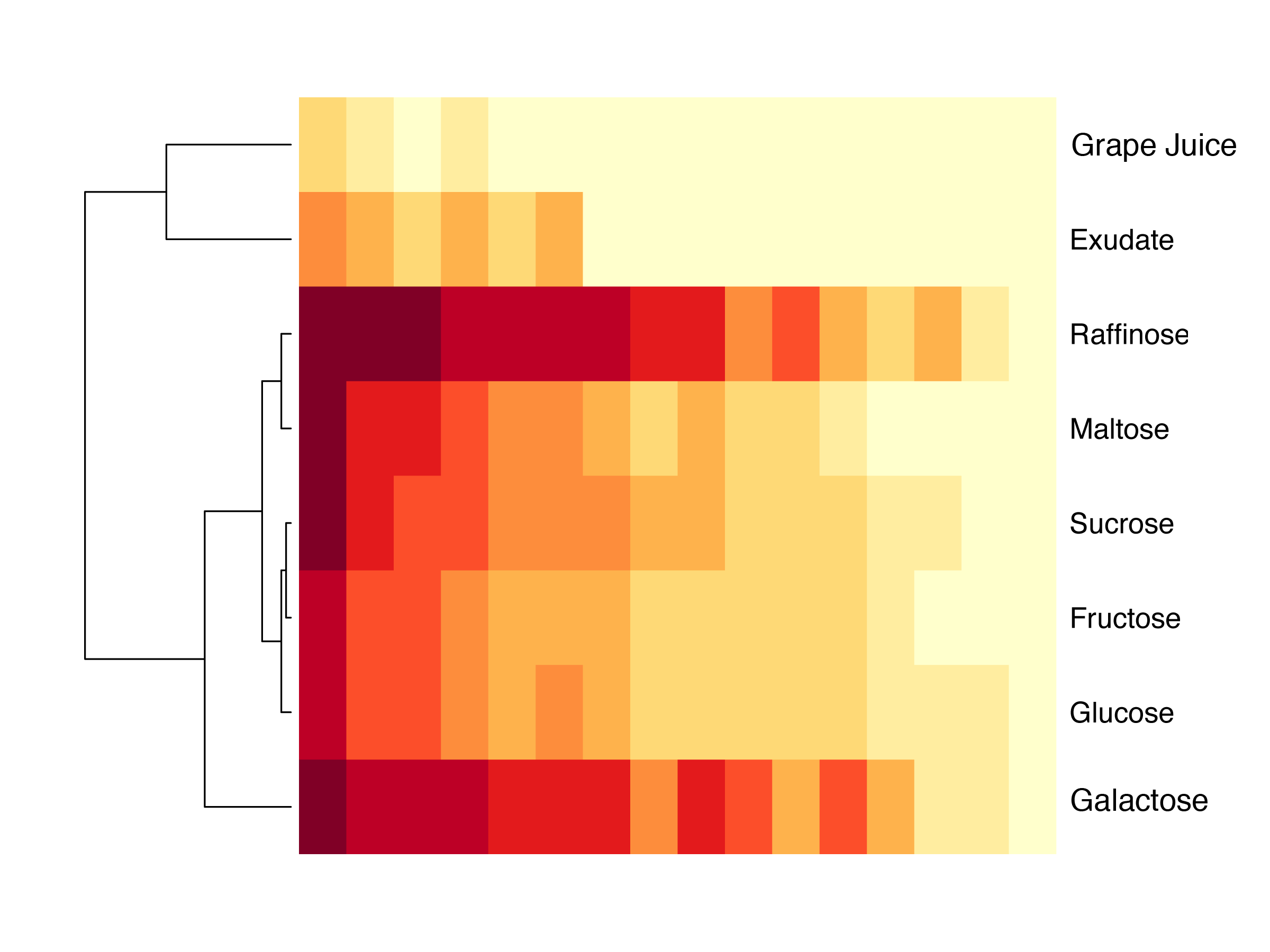

Supplement: Figure S2 — Heatmap produced by clustering sporulation efficiencies in the vineyard background only. (0.15 MB TIF) [file pgen.1001144.s003.tif]

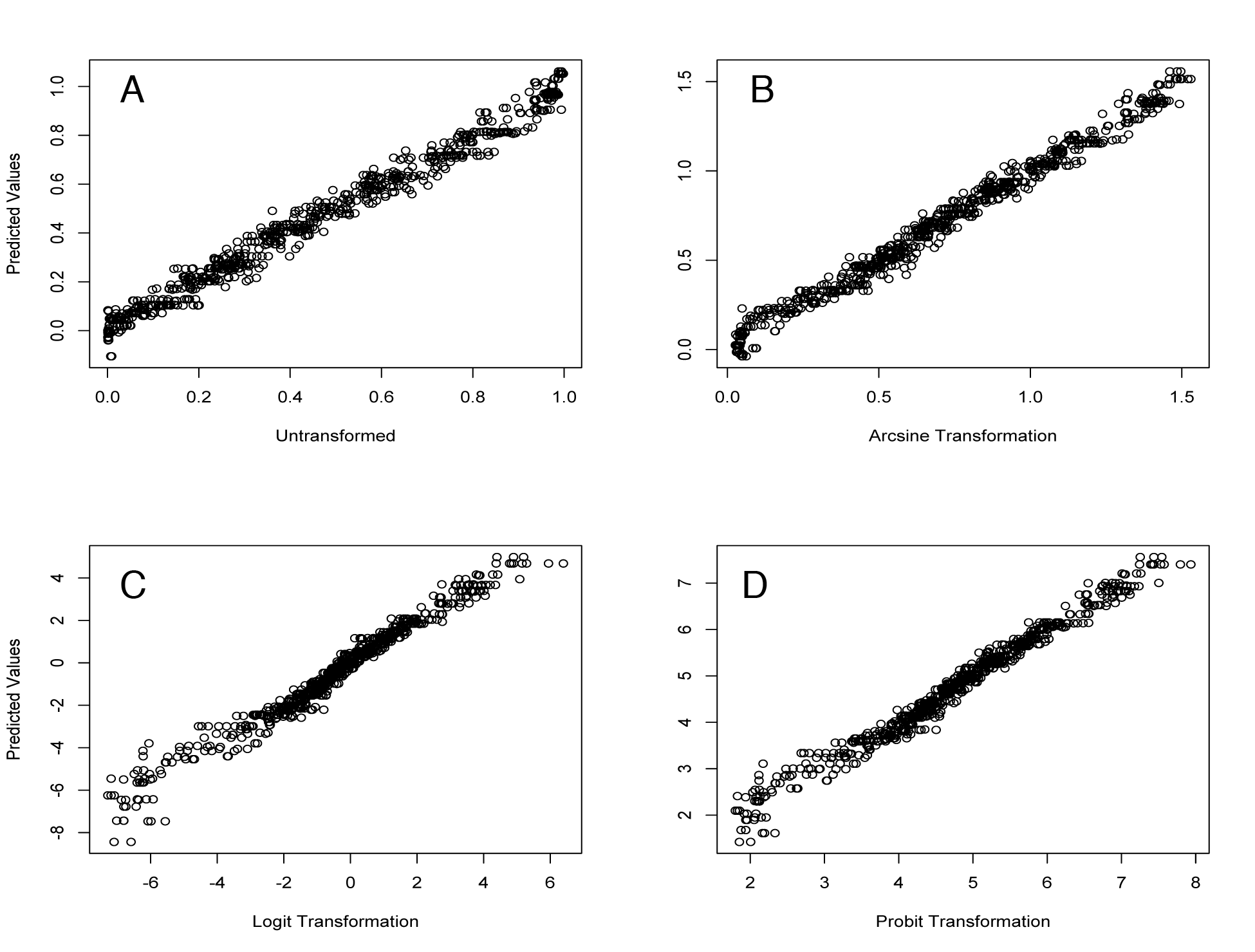

Supplement: Figure S3 — Three-way interaction models of sporulation efficiency after scale transformations. Actual values for each strain replicate are denoted on the x-axis, and the predicted values are on the y-axis. (A) The raw linear scale. (B) Arcsine transformation. (C) Logit transformation. (D) Probit transformation. These scale transformations reduced the number of interaction terms in models of some single environments. (0.21 MB TIF) [file pgen.1001144.s004.tif]
